# Exposure Medium: Key in Identifying Free Ag^+^ as the Exclusive Species of Silver Nanoparticles with Acute Toxicity to *Daphnia magna*

**DOI:** 10.1038/srep09674

**Published:** 2015-04-10

**Authors:** Mo-Hai Shen, Xiao-Xia Zhou, Xiao-Ya Yang, Jing-Bo Chao, Rui Liu, Jing-Fu Liu

**Affiliations:** 1 State Key Laboratory of Environmental Chemistry and Ecotoxicology, Research Center for Eco-Environmental Sciences, Chinese Academy of Sciences, P. O. Box 2871, Beijing 100085, China; 2 School of the Environment, Jiangsu University, Zhenjiang 212013, China; 3 Chemical Metrology and Analytical Science Division, National Institute of Metrology, Beijing 100013, China

## Abstract

It is still not very clear what roles the various Ag species play in the toxicity of silver nanoparticles (AgNPs). In this study, we found that traditional exposure media result in uncontrollable but consistent physicochemical transformation of AgNPs, causing artifacts in determination of median lethal concentration (LC50) and hindering the identification of Ag species responsible for the acute toxicity of AgNPs to *Daphnia magna*. This obstacle was overcome by using 8 h exposure in 0.1 mmol L^−1^ NaNO_3_ medium, in which we measured the 8-h LC50 of seven AgNPs with different sizes and coatings, and determined the concentrations of various Ag species. The LC50 as free Ag^+^ of the seven AgNPs (0.37–0.44 μg L^−1^) agreed very well with that of AgNO_3_ (0.40 μg L^−1^), and showed the lowest value compared to that as total Ag, total Ag^+^, and dissolved Ag, demonstrating free Ag^+^ is exclusively responsible for the acute toxicity of AgNPs to *D. magna*, while other Ag species in AgNPs have no contribution to the acute toxicity. Our results demonstrated the great importance of developing appropriate exposure media for evaluating risk of nanomaterials.

Due to their unique properties especially the excellent antibacterial capability, silver nanoparticles (AgNPs) are produced in large production and applied in various areas^1^,^2^,^3^,^4^. This leads to the release of AgNPs into the environments, and the wide detection of AgNPs together with their transformation products like nanoparticulate Ag_2_S in waste water treatment plants^5^. Therefore, the environmental processes and toxicity of AgNPs are more and more concerned.

Various studies on the toxicity of AgNPs, especially to aquatic organisms, were studied and well reviewed recently^1^,^2^,^3^,^4^. However, the mechanisms of the toxicity of AgNPs to organisms are not quite clear yet. Although some studies suggest that only the dissolved silver is responsible for the toxicity to *Daphnia magna*^6^,^7^, many results suggest that both the dissolved and nanoparticulate silver contribute to the toxicity of AgNPs, and Ag^+^ is the main source of direct toxicity^8^,^9^,^10^,^11^,^12^,^13^,^14^,^15^.

Since AgNPs are highly dynamic in aquatic systems, during the exposure they would undergo different chemical and morphology transformation, such as chemical oxidation of AgNPs to release Ag^+^, formation of second generation AgNPs from the released Ag^+^, and the aggregation/reconstitution of AgNPs, which would in turn greatly affect their bioavailability and toxicity^8^,^16^,^17^,^18^,^19^,^20^,^21^,^22^,^23^,^24^,^25^,^26^,^27^,^28^. Consequently, it is likely that in aquatic exposure systems, AgNPs are present in altered morphology, and co-existed with released Ag^+^ that may present in the forms of free ions and complexes with the capping agents or ligands in the exposure media, as well as adsorbed on the particles. It was reported that during the exposure of AgNPs to *D. magna*, the standard exposure media recommended by Organization for Economic Co-operation and Development (OECD) and Environmental Protection Agency (EPA) with salts like CaCl_2_ (CaSO_4_), MgCl_2_ (MgSO_4_) and Na_2_CO_3_ (NaHCO_3_) usually cause uncontrollable but consistent aggregation of AgNPs, and variation of silver species concentration due to the reaction of Ag^+^ with Cl^−^, SO_4_^2−^, CO_3_^2−^ in the culture media, and therefore gave rise to changes in organism exposure levels and the nature of the exposed particles compared to exposure to fully dispersed particles^8^,^24^,^29^. However, these very few studies focus on the physical effects, and the variation of chemical species of AgNPs in the exposure media remains unclear. Thus, to elucidate the toxicity mechanism of AgNPs to *D. magna*, it is of great importance to develop exposure media that are able to preserve the initial physical and chemical properties of AgNPs during the exposure, as well as to accurately determine the various Ag species in AgNPs^30^.

Currently, various methods have been developed to determine the optional species in AgNPs. Dialysis^17^ and flow field-flow fractionation^8^,^31^,^32^,^33^ was used to separate AgNPs from free Ag^+^ and low molecular weight (MW) ligand complexed Ag^+^. Centrifugal ultrafiltration (UF)^6^,^26^,^34^,^35^ and diffusive gradients in thin films (DGT)^36^ were adopted to separate AgNPs from dissolved Ag, which including free Ag^+^, ligand complexed Ag^+^, and tiny AgNPs (<2 nm). While ion-selective electrode (ISE) provides a inexpensive, sensitive and selective technique for direct measurement of free Ag^+ 36,37^, single-particle inductively coupled plasma mass spectrometry was adopted to directly quantify AgNPs with sizes ≥20 nm^38^,^39^. Cloud point extraction^5^,^40^,^41^,^42^,^43^,^44^, reversed-phase liquid chromatography (LC)^45^,^46^ and capillary electrophoresis (CE)^47^ were coupled with inductively coupled plasma mass spectrometry (ICP-MS) for speciation test of AgNPs and dissoluble Ag^+^, which was defined as including free Ag^+^, Ag^+^ complexed with ligands, Ag^+^ adsorbed on the AgNP surface, and originally undissolved silver salts that can be dissolved in the presence of Na_2_S_2_O_3_.

The objective of this work is to evaluate the contribution of various Ag species in AgNP exposure mixtures to the toxicity towards *D. magna*. To exclude the artifacts from the exposure media, we at first sought an exposure medium that was able to minimize the aggregation of AgNPs, eliminate the precipitation of Ag^+^, and ensure the living of *D. magna*. Then, the acute toxicity of AgNPs with varied coating and sizes were assessed using the optimized medium, and the median lethal concentration (LC50) in terms of total Ag, free Ag^+^, total Ag^+^, dissolved Ag, and nano Ag were determined with different analytical techniques. Finally, the concentration of various Ag species in exposure mixtures of different AgNPs were compared to LC50 of Ag^+^ as AgNO_3_, to identify which species of Ag is responsible for the AgNP acute toxicity towards *D. magna*.

## Results

### Characterization of AgNPs

A series of AgNPs varied in their primary size and surface coating were studied herein. Fig. S1 (of Supporting Information (SI)) shows that both the synthesized polyvinylpyrrolidones (PVP) coated AgNPs (herein referred to as AgNP_PVP_) and commercially obtained sodium-citrate coated AgNPs (herein referred to as AgNP_CIT_) were in spherical or near-spherical structures in ultrapure water. No visible coatings were evident in AgNP_PVP_ transmission electron microscopy (TEM) images, indicating the excess PVP in suspensions were effectively removed in wash procedures. Counted from over 133 particles in TEM images, the respective primary particle sizes of the two synthesized AgNP_PVP_ were 9.9 ± 2.2, and 28.1 ± 4.6 nm (herein referred to as AgNP_PVP10_ and AgNP_PVP28_), while that of the 5 commercial AgNP_CIT_ (herein referred to as AgNP_CIT10_, AgNP_CIT20_, AgNP_CIT40_, AgNP_CIT60_ and AgNP_CIT100_) were 9.2 ± 1.7, 20.7 ± 2.8, 40.0 ± 3.9, 56.3 ± 5.9, 95.5 ± 7.8 nm, respectively, agreed very well with their commercially reported sizes. All the studied AgNPs had a negative ζ-potential in ultrapure water, due to that PVP or citrate were used as stabilizing and coating material.

### Development of Acute Toxicity Testing Medium for *Daphnia magna*

**Figure 1:**
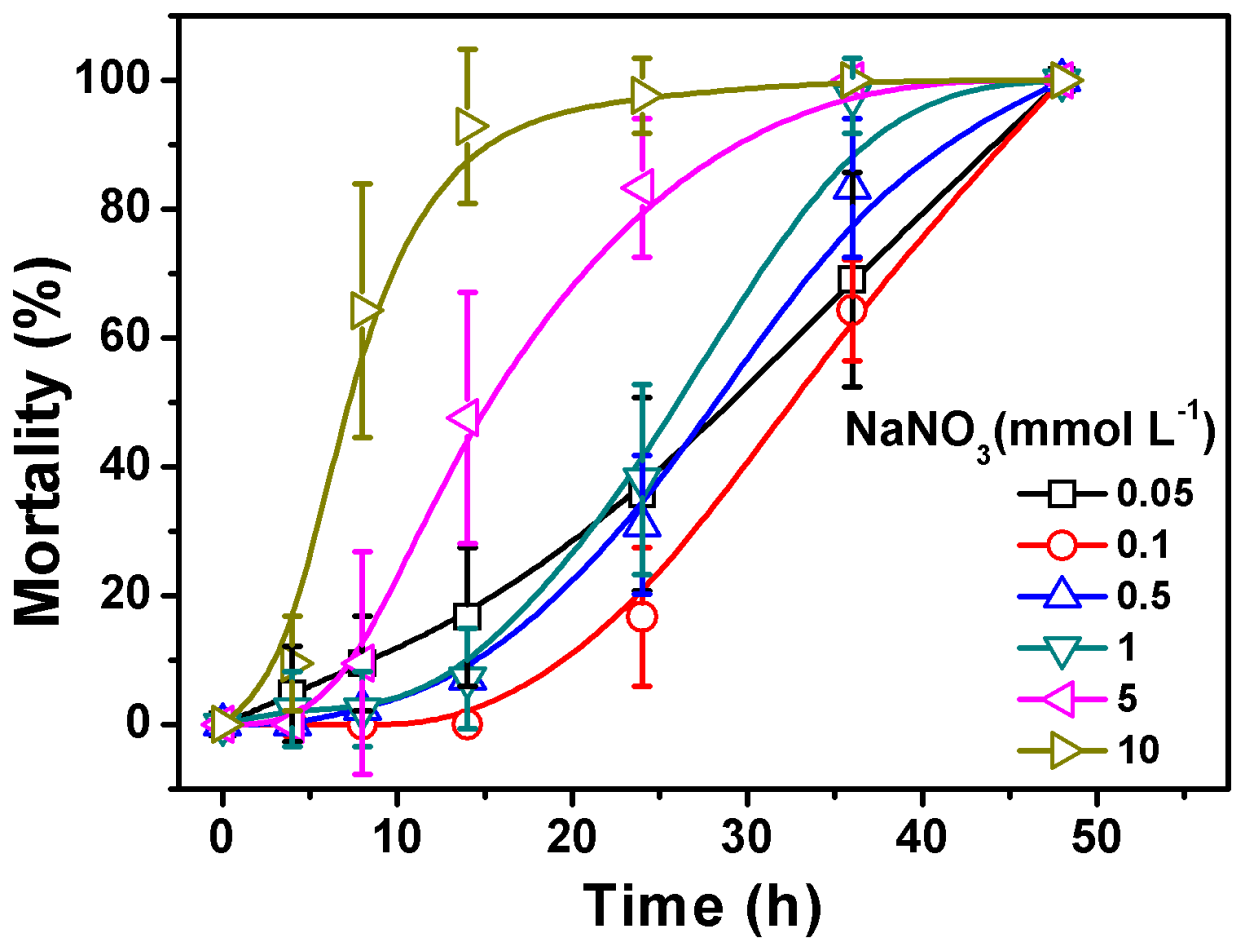
Development of acute toxicity testing medium for *Daphnia magna.* Mortality of *Daphnia magna* in NaNO_3_ solutions at concentration over the range of 0.05–10 mmol L^−1^ (pH 7.8 ± 0.2 adjusted by NaOH) in several time intervals during 48-h exposure. Error bars represent the standard deviation from 6 parallels.

The EPA and OECD standard exposure media with diverse ions were established with primary concerns for the living maintenance of *D. magna*. These media have long been applied for testing the acute toxicity of traditional chemicals, as they do not cause physicochemical changes to the tested chemicals. Given the various ions in EPA and OECD media could alter the state and speciation of AgNPs/Ag^+^ for their special properties, NaNO_3_ was selected to develop a medium that is able to maintain both the state of AgNPs/Ag^+^ and the living of *D. magna*, even though a medium containing single type of ion would not be favourable for the survival of *D. magna* as the EPA and OECD media did. The 48-h mortality experiments showed that with 0.1 mmol L^−1^ NaNO_3_ medium (pH 7.8 ± 0.2 adjusted by NaOH), the *D. magna* all lived well and showed normal behavior in at least 14 h without feeding, while with NaNO_3_ media at other studied concentrations the death of *D. magna* occurred within 8-h exposure, indicating the media with either higher or lower concentrations of NaNO_3_ were more toxic relative to 0.1 mmol L^−1^ NaNO_3_ upon *D. magna* (Fig. 1). The higher toxicity of media with higher concentration of NaNO_3_ would probably due to the toxicity of NO_3_^−^ to *D. magna*, whereas the more toxic of media with lower concentration of NaNO_3_ could be attributed to the insufficient ionic strength of the media to maintain the lives of *D. magna*. Thus, test of 8-h acute toxicity to *D. magna* in 0.1 mmol L^−1^ NaNO_3_ (pH 7.8 ± 0.2 adjusted by NaOH) medium was adopted as optimum. We acknowledge that the 8-h exposure was relatively short, but it was a compelled choice due to concerns over the survival assurance of *D. magna* in this specific exposure medium. It should also be noted that, as the aim of this study was to identify the relative importance of various Ag species in AgNP exposure mixtures to the acute toxicity towards *D. magna* by using a reliable exposure medium, the relative short term exposure would actually help understand the rapid response of *D. magna* to certain Ag species, thus reveal the Ag species with relatively high acute toxicity towards *D. magna*. In addition, it was found that some previous studies also chose short term exposure (≤8 h) in toxicity study to *D. magna* to meet special concerns^48^,^49^.

**Figure 2:**
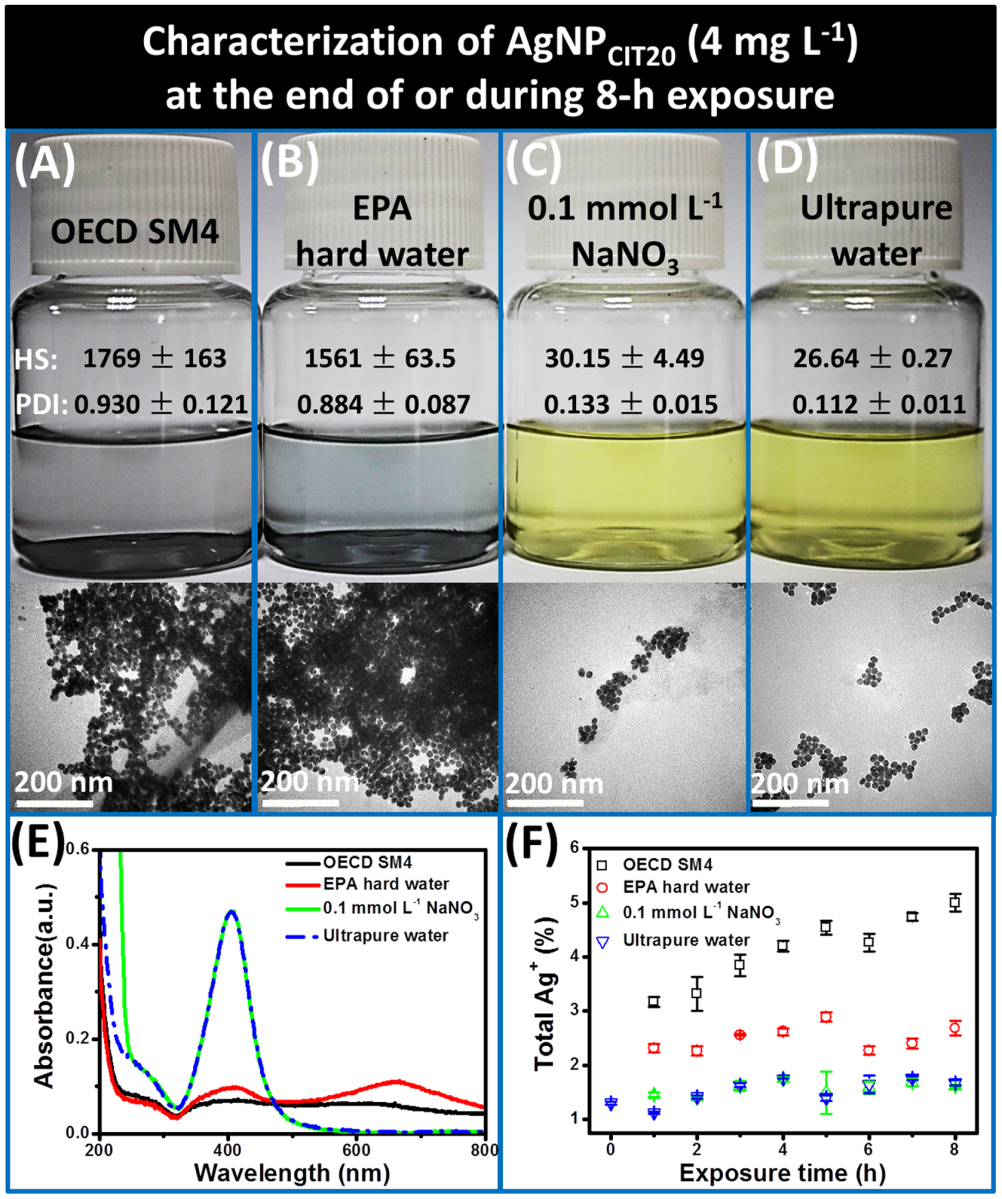
Characterization of AgNP_CIT20_ (4 mg L^−1^) at the end of or during 8-h exposure in different media. (A–D) Photograph, TEM images, hydrodynamic sizes (HS, nm) and polydispersity index (PDI) of AgNP_CIT20_ (4 mg L^−1^) in OECD SM4, EPA hard water, 0.1 mmol L^−1^ NaNO_3_ and ultrapure water at the end of 8-h exposure. (E) UV-vis spectra of AgNP_CIT20_ (4 mg L^−1^) in these media at the end of 8-h exposure. (F) Release of total Ag^+^ from AgNP_CIT20_ (4 mg L^−1^) in these media during 8-h exposure.

Figure 2 shows the characterization of a representative AgNP, AgNP_CIT20_, at 8 h after being spiked in four exposure media including OECD simplified M4 (SM4) medium, EPA hard water, 0.1 mmol L^−1^ NaNO_3_, and ultrapure water, respectively. While the OECD and EPA media induced the aggregations of AgNPs (hydrodynamic sizes and TEM results shown in Fig. 2A–D), suppressed the absorbance at 400 nm (Fig. 2E), and turned the suspension color to blue (photograph) relative to that in ultrapure water, the 0.1 mmol L^−1^ NaNO_3_ medium resulted in negligible change in comparison to that in ultrapure water. Fig. 2F also shows the total Ag^+^ released from these media during 8-h exposure. At the initial of exposure, the total Ag^+^ content accounted for only 1.3% of the total Ag in AgNP_CIT20_ (4 mg L^−1^ as total Ag). In OECD and EPA media, proportions of total Ag^+^ increased to 5.0% and 2.7%, respectively. Whereas, the total Ag^+^ elevated only a little to 1.6% in NaNO_3_ medium and to 1.7% in ultrapure water at the end of 8-h exposure. The analysis of variance (ANOVA) results further indicated that the total Ag^+^ release from the later two media had no significant differences either during (*p* = 0.922) or at the end of (*p* = 0.116) 8-h exposure. These evidence suggested that, in the NaNO_3_ medium, although slight increase of Ag^+^ content appeared as the exposure proceeded from 0 to 8 h due to the inevitable reactions with dissolved oxygen^16^, the Ag^+^ content of AgNP suspension during acute toxicity exposure test was stabilized to the greatest extent as in ultrapure water and showed no significant changes during 8-h exposure (*p* = 0.06). These combined evidences in Fig. 2 indicate that the 0.1 mmol L^−1^ NaNO_3_ medium could significantly retard the physicochemical transformation of AgNPs relative to the OECD and EPA media recommended for *D. magna* acute toxicity exposure.

### Median Lethal Concentration of AgNPs to *Daphnia magna* in 0.1 mmol L^−1^ NaNO_3_ Medium

**Figure 3:**
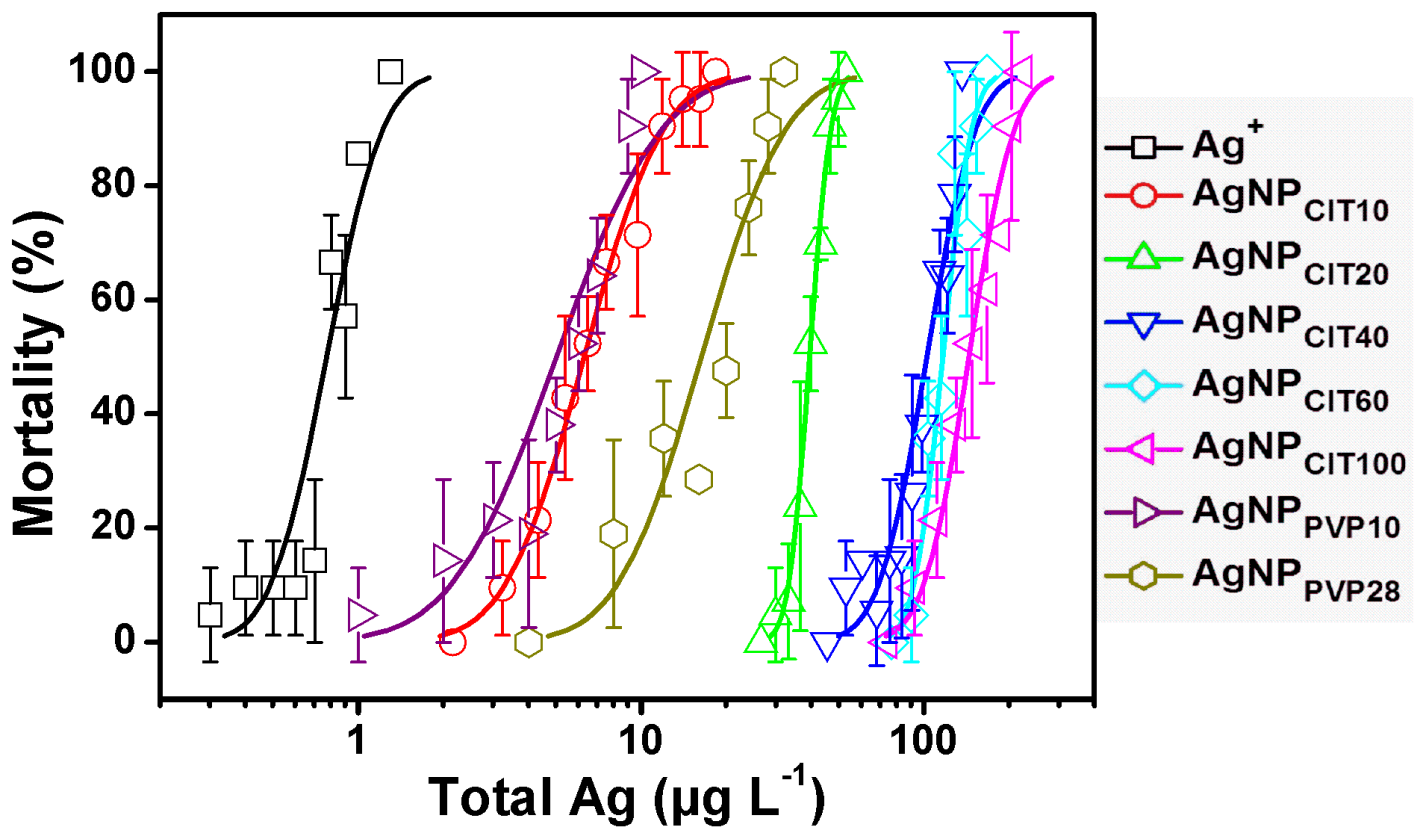
Dose-response curves of mortality of *Daphnia magna* against exposure concentration as nominal total Ag of AgNO_3_ and AgNPs. The AgNO_3_ and AgNPs suspensions (five sodium-citrate coated AgNPs (AgNP_CIT_) and two polyvinylpyrrolidones (PVP) coated AgNPs (AgNP_PVP_)) were all prepared in 0.1 mmol L^−1^ NaNO_3_ (pH 7.8 ± 0.2 adjusted by NaOH) medium. Error bars represent the standard deviation from triplicates. The lethal concentration probability lines were plotted on the basis of probit regression results obtained from SPSS 16.0 software.

The mortality values of *D. magna* against exposure concentrations of AgNO_3_ and AgNPs were quantitatively presented in Fig. 3, with lethal concentration probability lines obtained from probit regressions by SPSS 16.0 software on the basis of the original data. The median lethal concentration for 8-h (LC50_8-h_) as nominal total Ag showed that toxicity of AgNPs spanned an order of magnitude with values varying in the range of 5.04 to 144.25 μg L^−1^, and AgNO_3_ was much acute toxic than AgNPs with LC50_8-h_ of 0.78 μg L^−1^ (Table S1 of SI). For AgNO_3_, the LC50_8-h_ agreed with the 48-h LC50, which was reported as 0.58–2.51 μg L^−1^ as nominal total Ag in literature^6^,^7^,^10^,^11^,^50^,^51^,^52^.

### Measured Concentrations of Ag Species in AgNP Suspensions Equivalent to LC50_8-h_

With the analytical methods shown in Table 1, we determined the concentrations of total Ag, free Ag^+^, total Ag^+^ and dissolved Ag in solutions/suspensions equivalent to LC50_8-h_ of AgNO_3_ and AgNPs (Table S2 of SI), and further calculated the nano Ag concentration and the proportion of Ag species in total Ag (Table S3 of SI). The measured total Ag at LC50_8-h_ were 4.68–126.51 μg L^−1^ for AgNPs and was 0.44 for AgNO_3_ (Table S2 of SI), which were generally lower than their corresponding nominal total Ag concentration (Table S1 of SI). This was ascribed to the adsorption of a fringe of Ag species to glass-beakers during the exposure. The LC50_8-h_ as measured total Ag would be more persuasive than that as nominal total Ag. Hereafter, the total Ag referred to as the measured total Ag in suspension equivalent to LC50_8-h_ condition, unless otherwise stated. For AgNPs with similar size but different coatings, the measured total Ag at LC50_8-h_ for AgNP_PVP_ appeared to be relevantly lower than that for AgNP_CIT_ (*e.g.* 14.01 μg L^−1^ for AgNP_PVP28_ *vs* 36.25 μg L^−1^ for AgNP_CIT20_). In addition, as the size of AgNPs (with same coating) increased, the total Ag increased as well (Table S2 of SI). For instance, converting the AgNP_CIT10_ to AgNP_CIT100_, total Ag concentrations increased from 5.70 to 126.51 μg L^−1^.

The LC50_8-h_ as free Ag^+^ concentration for AgNO_3_ was 0.40 ± 0.05 μg L^−1^. As the measured total Ag should be equivalent to its free Ag^+^ for AgNO_3_, we performed the ANOVA to test on them. The result showed no significant difference between the two measured values (*p* = 0.27, Table S4 of SI), thus partially proved the credibility of free Ag^+^ results determined by ISE method. LC50_8-h_ as free Ag^+^ concentrations in seven AgNPs were almost constant with values in range of 0.37–0.44 μg L^−1^ (Table S2 of SI). The ANOVA results showed that the free Ag^+^ contents in AgNP suspensions had no significant differences against coatings or sizes of AgNPs (*p* = 0.91, >0.05, Table S4 of SI). However, they accounted for wide-range proportions of 0.32% to 9.31% of the measured total Ag, and the proportion increased significantly as the size of AgNP increased (Table S3 of SI).

Concentrations of total Ag^+^ and dissolved Ag were relatively higher than that of free Ag^+^ in identical AgNP suspension, with concentration ranges of 1.05–2.62 μg L^−1^ and 0.91–1.58 μg L^−1^, respectively. Although LC50_8-h_ as total Ag^+^ and dissolved Ag both differed significantly among AgNP suspensions (*p* ≪ 0.05, Table S4 of SI), neither of them showed size-dependent changes among AgNPs.

Compared with the above Ag species, the nano Ag was the primary portion in AgNP suspension. For AgNPs with the same coating, the proportion of nano Ag generally increased with the size of AgNPs. Take AgNP_CIT_ for instance, the proportion of nano Ag increased from 81.57% to 98.30% as the size of AgNP_CIT_ elevated from 10 nm up to 100 nm.

### Relevance of Different Silver Species to the Toxicity towards *Daphnia magna*

**Figure 4:**
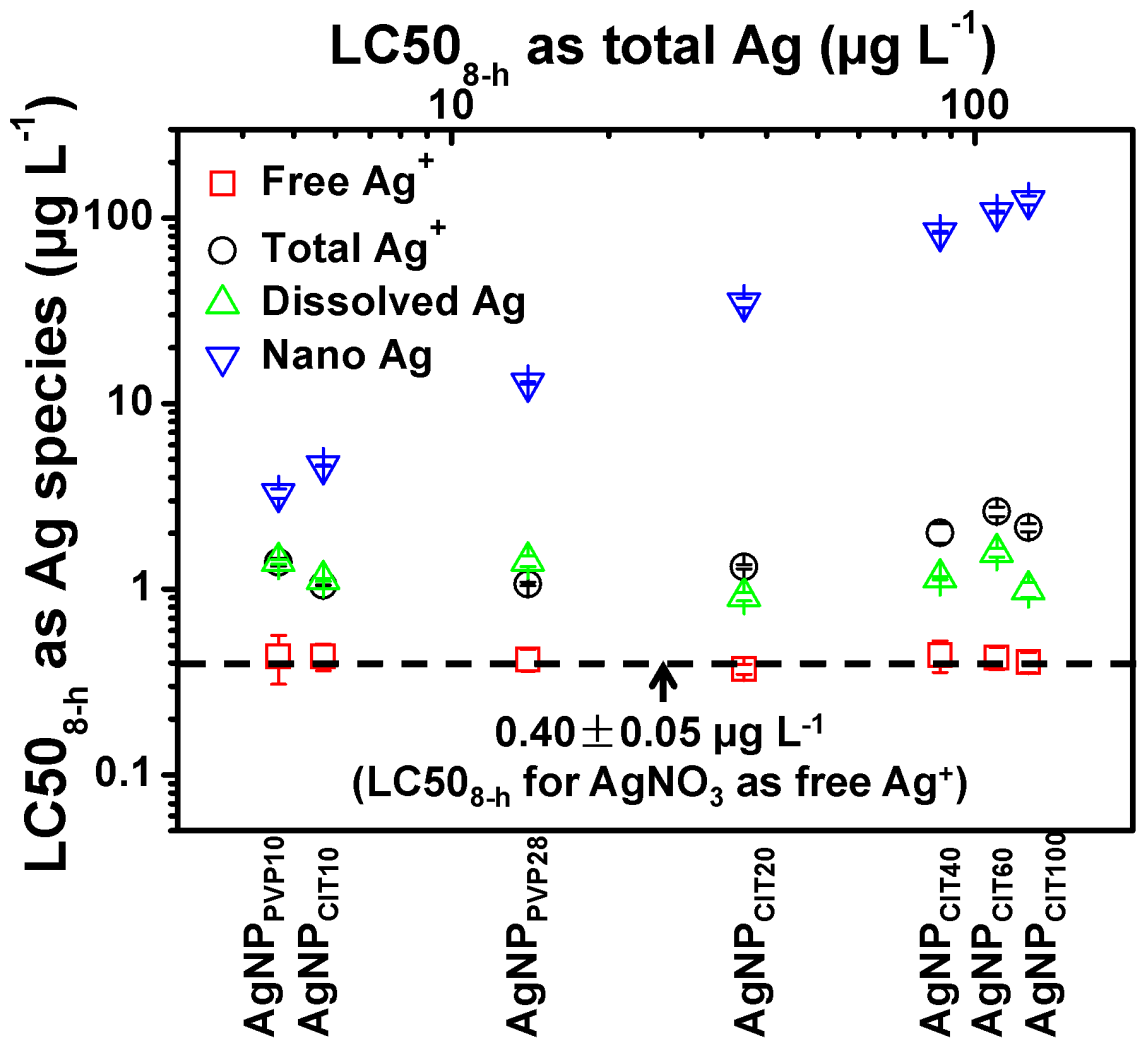
LC50_8-h_ as different Ag species in AgNP suspensions. LC50_8-h_ as free Ag^+^, total Ag^+^, dissolved Ag and nano Ag concentrations plotted against that as measured total Ag in all AgNP suspensions equivalent to LC50_8-h_ suspensions. Dash line indicates LC50_8-h_ for AgNO_3_ (as free Ag^+^).

With the above measured concentration of various Ag species in AgNP suspensions equivalent to LC50_8-h_, we can present the LC50_8-h_ values in terms of different Ag species. As shown in Fig. 4, for the seven tested AgNPs, while the LC50_8-h_ as nano Ag spanned over an order of magnitude, the LC50_8-h_ as different dissolved Ag species were relatively close to that as Ag^+^ in AgNO_3_. To identify the most responsible Ag species to the toxicity of AgNP suspension towards *D. magna*, ANOVA was performed to test the significances of concentrations of different dissolved Ag species among AgNPs with that from the LC50_8-h_ values for AgNO_3_ (Table S4 of SI). First, the free Ag^+^ concentrations in AgNP suspensions at their LC50_8-h_ concentration levels had no significant differences from the measured free Ag^+^ LC50_8-h_ for AgNO_3_ (0.40 μg L^−1^ as Ag^+^) either (*p* = 0.66, >0.05). For concentrations of total Ag^+^ and dissolved Ag, however, the ANOVA results showed that the two Ag species had significant difference from the measured free Ag^+^ LC50_8-h_ for AgNO_3_ (*p* ≪ 0.05).

## Discussion

In traditional exposure media, a critical obstacle in the toxicity study of AgNPs was the occurrence of uncontrollable but consistent physical and chemical transformation (*e.g.* aggregation and dissolution) of AgNPs, due to the high chemical reactivity of Ag^0^ with dissolved O_2_, and Ag^+^ with anions like Cl^−^. In addition, the relatively high ionic strength of the exposure media also lead to physical changes of AgNPs, which in turn affects the dissolution of AgNPs to release Ag^+^. Therefore, the measured concentrations of Ag species in traditional media at an exact time point cannot profile the concentration during the entire exposure period (as shown in Fig. 2F). However, currently most of the reported LC50 of different Ag species to *D. magna* or other aquatic organisms in OECD/EPA media were related to concentrations of corresponding Ag species at the initial or end point of 24-h or 48-h exposure, which inevitably causes large artifacts in the measured LC50 values due to the significant changes of state and speciation of AgNPs/Ag^+^, regardless they were presented as total Ag, dissolved Ag, or free Ag^+^. Consequently, previous studies have reported mixed results: some show dissolved Ag is responsible for the AgNP toxicity^6^,^7^,^9^, others show free Ag^+^ predominant toxicity^10^,^12^ and particle-specific toxicity^13^,^14^.

In this study, by using 8-h exposure in 0.1 mmol L^−1^ NaNO_3_ medium, the uncertainty of physical changes of AgNPs and their influences on the dissolution of AgNPs during exposure were significantly excluded. The ANOVA results on Ag^+^ contents in the representative AgNP_CIT20_ suspension further showed that, the Ag^+^ contents during the 8-h exposure in the 0.1 mmol L^−1^ NaNO_3_ medium had no significant changes (*p* = 0.06, >0.05), suggesting the optimized exposure medium in the present study could significantly retard the dissolution and oxidation of AgNPs to Ag^+^ during the 8-h exposure procedure. Therefore, this more reliable exposure medium ensured the quantification of Ag species in solutions equivalent to the LC50_8-h_ conditions, and the identification of the relative importance of various Ag species in AgNP exposure mixtures to the toxicity towards *Daphnia magna*. This excluded the artifacts in evaluation of LC50, thus unlike the wide span of LC50 value as free Ag^+^ (0.57–1.1 μg L^−1^) reported in a previous study^12^, almost the same LC50 value (0.37–0.44 μg L^−1^) as free Ag^+^ was obtained for 7 different AgNPs in this study.

Based on the LC50 values (Fig. 4) that was accurately measured with the proposed exposure procedure, it is possible to weight the actual contribution of different Ag species to the toxicity of AgNPs, and therefore distinguish the intrinsic Ag species responsible for the acute toxicity of AgNP suspension to *D. magna*.

For all the tested seven AgNPs with different coatings and sizes, the values of LC50_8-h_ as free Ag^+^ were very close to that of AgNO_3_, and were lower than that as total Ag^+^ and as dissolved Ag. Given that the total Ag^+^ covered the free Ag^+^, Ag^+^ complexes and absorbed Ag^+^ on AgNP surfaces, and the dissolved Ag contained free Ag^+^, Ag^+^ complexes and tiny AgNPs, these results thus indicated that the other parts except for free Ag^+^ showed no contribution to the acute toxicity, namely free Ag^+^ is the exclusive contributor of acute toxicity to *D. magna*. If the measured LC50 as total Ag^+^ or dissolved Ag had been lower than that as free Ag^+^, it would have indicated that the AgNP adsorbed Ag^+^, ligand complexed Ag^+^, and tiny AgNPs contributed to the observed toxicity.

To aquatic organisms, it is generally believed that free metal ions are bioavailable, while other metal species such as metal ions complexed with organic ligands, and associated to dissolved organic matter or particles are not bioavailable^53^,^54^,^55^. Since AgNPs were reported to be directly available, it is controversial if AgNPs showed particle effects to the acute toxicity to organisms. Our results suggest that particulate AgNPs, even tiny AgNPs (<2 nm) that was found to present in the dissolved Ag (Fig. S2 of SI), showed negligible contribution to the acute toxicity to *D. magna*. Although the 8-h acute toxicity exposure performed in the present study was relatively shorter than that in traditional experiments, it in fact proved that the free Ag^+^ showed highly acute toxicity towards *D. magna* at a very short term relative to any other Ag species. It is noteworthy that this is the first study elucidating free Ag^+^ is exclusively responsible for the acute toxicity of AgNPs to *D. magna*, though previous studies reported that free Ag^+^ dominate the toxicity of AgNPs to bacteria^34^,^56^.

Numerous studies have reported the influence of size and coating on the toxicity of AgNPs. Difference of 48-h LC50 values among three AgNPs with different coatings^12^ and varied sizes^7^ were reported. The present study showed that the acute toxicity of AgNPs to *D. magna* represented as total Ag decreased in the order of AgNO_3_ ≫ AgNP_PVP_ > AgNP_CIT_, and the toxicity of AgNP_CIT_ decreased with the increase of size. Specifically, the LC50_8-h_ as total Ag among AgNPs with different sizes and surface coatings varied by a factor of 27. Apparently, this was probably due to the changes of dissolution of different types of AgNPs as influenced by nanoparticle size and surface-coating. For AgNPs with the same coating, the proportion of total Ag^+^ generally decreased with the increasing size of AgNPs. Take AgNP_CIT_ for instance, the proportion of total Ag^+^ decreased from 18.43% to 1.70% as the size of AgNP_CIT_ elevated from 10 nm to 100 nm. This was in accordance with expectation since the increase of AgNP particle size reduced the relative surface area, thus retarded the release of dissolved Ag species, and exhibit lower toxic responses^20^. For AgNP_PVP_ and AgNP_CIT_ with similar sizes, the proportion of total Ag^+^ for AgNP_PVP_ was generally higher than that of AgNP_CIT_, which could be attributed to the reducibility of sodium-citrate relatively retarded the dissolution of Ag^0^ to Ag^+^ 17. However, the LC50_8-h_ as free Ag^+^ showed no significant difference among AgNPs and agreed very well with that of AgNO_3_. Together, our results indicate free Ag^+^ was the intrinsic and ultimate reason that governs the acute toxicity of AgNPs, while size and surface coating are apparent factors that influence the toxicity through affecting the free Ag^+^ concentration of AgNPs.

In summary, uncontrollable but consistent physical and chemical transformation of AgNPs occurred in traditional OECD and EPA exposure media, which hinders the identification of Ag species of AgNPs responsible for the acute toxicity to *D. magna*. By using 8 h exposure in 0.1 mmol L^−1^ NaNO_3_ medium, the artifacts in determination of LC50 were excluded, and the LC50_8-h_ as free Ag^+^ of all the seven AgNPs were found to be almost the same and agree with that of AgNO_3_. More importantly, with this developed exposure procedure, we found that free Ag^+^ is exclusively responsible for the acute toxicity of AgNPs to *D. magna*, while other Ag species in AgNPs like Ag^+^ complexes, Ag^+^ absorbed on AgNP surfaces, and nanoparticulate Ag showed no contribution to the acute toxicity. Our results also showed that free Ag^+^ was the intrinsic and ultimate factor that governs the acute toxicity of AgNPs, while size and surface coating are apparent factors that influence the toxicity through affecting the free Ag^+^ concentration of AgNPs.

## Methods

### Organisms, Chemicals and Materials

*D. magna* was obtained from Chinese Center for Disease Control and Prevention (Beijing, China), cultured in the charcoal-filtered tap water at 25°C and 16:8 h light: dark cycle and fed daily with green algae *Scenedesmus quadricauda*. *S. quadricauda* were cultured in Shuisheng NO. 4 medium (containing (NH_4_)_2_SO_4_ 0.15 mmol L^−1^, Ca(H_2_PO_4_)_2_ 0.03 mmol L^−1^, CaSO_4_ 0.40 mmol L^−1^, MgSO_4_ 0.32 mmol L^−1^, NaHCO_3_ 1.19 mmol L^−1^, KCl 0.34 mmol L^−1^, FeCl_3_ 0.03 mmol L^−1^ and soil-extract 0.5 mL) at 25°C and 16:8 h light: dark cycle. Commercially available AgNP_CIT_ (AgNP_CIT10_, AgNP_CIT20_, AgNP_CIT40_, AgNP_CIT60_ and AgNP_CIT100_) were purchased from Sigma-Aldrich (St. Louis, MO). PVP with molecular weight of 10,000 Da (PVP10,000) and 58,000 Da (PVP58,000) were purchased from Sigma-Aldrich and Aladdin Chemistry (Shanghai, China), respectively. The FL-70 was obtained from Fisher Scientific (Fair Lawn, NJ). Silver nitrate (AgNO_3_), sodium thiosulfate (Na_2_S_2_O_3_), sodium borohydride (NaBH_4_), and other salts were analytical-reagent grade or above and obtained from Sinopharm Chemical Reagent Beijing (Beijing, China). Ethylene glycol (99^+^% extra pure) was purchased from Acros Organics (Geel, Belgium). Nitric acid (65%) was obtained from Merck (Darmstadt, Germany). Amicon Ultra-15 centrifugal filter (with nominal molecular weight cut-off of 30 kDa) were obtained from Millipore (Darmstadt, Germany). All reagents were used without further purification. Ultrapure water (18 MΩ cm) produced from a Millipore Milli-Q Gradient system (Billerica, MA) were used to prepare all solutions throughout experiments.

### Synthesis and Characterization of AgNPs

Besides the 5 commercial AgNP_CIT_, another two AgNP_PVP_ (AgNP_PVP10_ and AgNP_PVP28_) were used in the present study. The two AgNP_PVP_ were synthesized following methods from published works^57^,^58^, and details were shown in SI.

### Characterization and Quantification of AgNPs

The TEM observation was preformed on JEM-2100F (JEOL) (see details in SI). The concentrations of AgNPs were quantified by ICP-MS (Agilent 7700, Santa Clara, CA), after room temperature HNO_3_ digestion as described in literature^46^. All the AgNP stock suspensions were kept in the dark at 4°C.

### Optimization of Acute Toxicity Testing Medium

To exclude the potential aggregation, precipitation and complexation of AgNPs deriving from the artifact of exposure media, NaNO_3_ was selected as a candidate for acute toxicity exposure media for AgNPs. To optimize the concentration of NaNO_3_ for acute toxicity experiment, the mortality of seven *D. magna* (6–24 h) in 50 mL media with different NaNO_3_ concentrations (0.05, 0.1, 0.5, 1, 5, 10 mmol L^−1^) was monitored at several time intervals during 48 h. The *D. magna* were not fed in the duration. All the prepared media were aerated for at least 24 h before exposure to *D. magna*, and the final pH of the media was all adjusted to 7.8 ± 0.2 using NaOH. The additional ionic strength by NaOH was no more than 0.05 mmol L^−1^, thus was assumed to be no additive effect on *D. magna* and AgNPs. Mortality experiments at each concentration point were repeated six times, and mortalities in each sample were assessed at time intervals of 4, 8, 14, 24, 36 and 48 h. Finally, the 0.1 mmol L^−1^ NaNO_3_ medium (pH 7.8 ± 0.2 adjusted by NaOH) was selected for a 8-h acute toxicity of AgNPs to *D. magna*.

### Characterization of AgNPs in Different Exposure Media

To compare the effect of the optimized exposure medium with traditional media on the stability of AgNP suspensions, a representative AgNPs (AgNP_CIT20_, 4 mg L^−1^ as total Ag) were prepared in OECD SM4 medium (which included most components of M4^59^ but excluded CoCl_2_, KI, Na_2_SeO_3_, NH_4_VO_3_ and Vitamins), EPA hard water^60^, 0.1 mmol L^−1^ NaNO_3_ medium and ultrapure water, respectively. The total Ag^+^ release from AgNP_CIT20_ in these media were monitored during the 8-h exposure. After 8-h exposure, the characterizations of AgNP_CIT20_, including UV-vis spectra (UV-3600 Spectrometer, Shimadzu, Japan), TEM imaging (preformed on H-7500 (Hitachi)), hydrodynamic sizes (Dynamic Light Scattering, DLS, Malvern, UK) and photographing of these samples were performed. TEM samples were prepared by loading 10 μL aliquots of suspensions onto ultrathin carbon-coated copper grid, and drying at room temperature under vacuum.

### Acute Toxicity Test of AgNPs to *Daphnia magna*

For acute toxicity test, seven *D. magna* (6–24 h old) were placed in 50-mL glass beakers containing 50 mL aliquots of the optimized NaNO_3_ exposure medium containing different concentrations (as total Ag) of each AgNPs. Triplicates of seven (or more) concentrations and medium-only control were tested for Ag^+^ (in AgNO_3_) and each AgNPs. Exposures were conducted at ~25°C in light for 8 h without feeding *D. magna*. Mortalities were assessed at the end of 8-h exposure. The LC50_8-h_, including 95% confidence intervals, were determined using SPSS 16.0 software applying the probit regression. The calculated LC50_8-h_ represented the nominal total Ag concentration, and concentration range of lethal effect (1% and 99%) were presented in Table S1 of SI.

### Determination of Ag Species in AgNP Suspension

The Ag species measurements, including total Ag, free Ag^+^, total Ag^+^ and dissolved Ag, were all conducted in new suspensions equivalent to the LC50_8-h_ of AgNO_3_ and AgNPs (as nominal total Ag), which were all prepared in 0.1 mmol L^−1^ NaNO_3_ medium. Definition and analytical methods used for the Ag species were listed in Table 1. The detailed determination procedures are as follows:

#### Determination of Total Ag by ICP-MS after Digestion

Concentrations of total Ag in suspensions equivalent to LC50_8-h_ (as nominal total Ag) of AgNO_3_ and AgNPs were measured using ICP-MS after room temperature HNO_3_ digestion^46^,^61^. Briefly, 0.5 mL AgNP sample was first digested with 0.5 mL 65% HNO_3_ for 15 min, and then diluted and mixed with 10 mL ultrapure water for ICP-MS measurement. The digestion-quantification procedure was repeated in triplicates for each sample. The calibration curve (with linear correlation coefficient, *R*^2^ > 0.999) was obtained from a series of standards of 0.1, 0.2, 0.5, 1, 2, 5, 10 and 20 μg L^−1^ Ag^+^ prepared in 5% HNO_3_.

#### Determination of Free Ag^+^ by ISE

Concentrations of free Ag^+^ in AgNPs at LC50_8-h_ (as nominal total Ag) were measured using a silver/sulfide ISE (Van-London pHoenix, Houston, TX). The standards for ISE measurement, with nominal concentrations in range of 0.2 to 5.0 μg L^−1^ of Ag^+^ (in AgNO_3_), were prepared in 0.1 mmol L^−1^ NaNO_3_ medium in 50-mL glass beakers. Given the predictable sorption of Ag^+^ to glass beakers after the preparation standard solutions would result in deviations of calibration, the Ag^+^ concentrations of ISE standards were determined by ICP-MS simultaneously with the ISE standards’ measurement (Fig. S3 of SI). The ICP-MS detected concentrations (in range of 0.18–3.99 μg L^−1^) were plotted against potential differences to prepare the calibration curve of ISE for measuring the free Ag^+^ in samples of AgNPs and AgNO_3_. A linear calibration obtained over the whole concentration range with slope of 50.7 mV was represented in Fig. S4 of SI. Free Ag^+^ measurements for each AgNP suspension were conducted at least in triplicates. The electrode was carefully washed by ultrapure water after each measurement, and dried off by dust-free paper prior to the next measurement.

#### Determination of Total Ag^+^ by LC-ICP-MS

Concentrations of total Ag^+^ in AgNPs at LC50_8-h_ (as nominal total Ag) were measured using on-line coupled LC (Agilent 1200, Santa Clara, CA) and ICP-MS (Agilent 7700). This LC-ICP-MS was recently established by our group for speciation analysis of nanoparticulate Ag (1–100 nm) from dissoluble Ag^+^, which was defined to includes free Ag^+^, Ag^+^ complexed with ligands, Ag^+^ adsorbed on the AgNP surface, and originally undissolved Ag(I) salts that can be dissolved in the presence of Na_2_S_2_O_3_^46^. For the simple AgNP exposure mixture used in this study, the dissoluble Ag^+^ equals to the total Ag^+^. Briefly, the LC separation of total Ag^+^ from the AgNP sample (20 μL) was performed with an Venusil Durashell-NH_2_ amino column (250 × 4.6 mm I.D., 5 μm particle size, 500 Å pore size, Bonna-Agela Technologies Inc., Tianjin, China) and a mobile phase of 0.1% (v/v) FL-70 (a surfactant) and 2 mmol L^−1^ Na_2_S_2_O_3_ in ultrapure water at a flow rate of 0.7 mL min^−1^. The total Ag^+^ was quantified by the coupled ICP-MS system. For the measurements of total Ag^+^ in AgNPs at LC50_8-h_, into the AgNP samples were added 2 mmol L^−1^ Na_2_S_2_O_3_ just before injection into the LC-ICP-MS system, with each sampling conducted in triplicates. The calibration curve prepared by the AgNO_3_ standards of 0.1, 0.2, 0.5, 1, 2 and 5 μg L^−1^ Ag^+^ in 2 mmol L^−1^ Na_2_S_2_O_3_ shows good linear correlation of *R*^2^ > 0.997. Given the excellent recovery of 87.4 ± 5.2% for total Ag^+^^46^, the total Ag^+^ values determined by LC-ICP-MS method in this study were without recovery correction.

#### Determination of Dissolved Ag by Centrifugal Ultrafiltration and ICP-MS

The concentrations of dissolved Ag in AgNPs at LC50_8-h_ (as nominal total Ag) were measured by centrifugal ultrafiltration off-line coupled with ICP-MS (UF&ICP-MS). The separation of dissolved Ag from AgNPs followed the method in our previous work^27^, which was modified from literature reported by Li and Lenhart^21^. Briefly, 10 mL aliquot of prepared sample was added to centrifugal ultrafilter devices (Amicon Ultra-15, 30 kDa), and the dissolved Ag was separated from AgNPs after centrifugation at 5000 rpm for 20 min. The dissolved Ag in the filtrate was collected and mixed with 0.2 mL 65% HNO_3_ for ICP-MS analysis. The quantification was conducted with calibration curve of Ag^+^ ranging from 0.1 to 5 μg L^−1^ with *R*^2^ > 0.997. The recovery of Ag through the 30-kDa membrane was 66.6 ± 14.6%, obtained by testing 5 mL AgNO_3_ solution at concentrations of Ag^+^ as 1.0 and 5.0 μg L^−1^ in 0.1 mmol L^−1^ NaNO_3_ with the same sample preparation procedure. The dissolved Ag values presented here were all with recovery correction, and each measurement was conducted in triplicates.

To test the existence of tiny particles in dissolved Ag, 30-kDa centrifugal ultrafiltration off-line coupled with LC-ICP-MS were performed on AgNP_PVP10_ at appropriate concentration. Fig. S2 of SI shows the presence of tiny particles in the filtrate of AgNP with particle size of ~1 nm, which was estimated according to the retention times of AgNP with different sizes following our previous method^46^.

## Author Contributions

M.H.S., X.X.Z., X.Y.Y., J.B.C. and R.L. performed the experiments. M.H.S. and J.F.L. analyzed the data and wrote the manuscript with revisions from all authors. J.F.L. conceived the research. All authors have given approval to the final version of the manuscript.

## Supplementary Material

Supplementary Information Supplementary Information

## Figures and Tables

**Table 1:** Definition and Quantification Method of the Studied Ag Species in AgNP Exposure Media

Ag species defined	Ag forms included	quantification method
Total Ag	All forms of Ag in suspension	ICP-MS after digestion
Free Ag^+^	Freely dissolved Ag^+^	Ion-selective electrode (ISE)
Total Ag^+^	Free Ag^+^ Ag^+^ complexed with ligands Ag^+^ adsorbed on the AgNP surface	LC-ICP-MS
Dissolved Ag	Free Ag^+^ Ag^+^ complexed with ligands Tiny AgNPs (<2 nm)	ICP-MS after centrifugal ultrafiltration with 30-kDa filter (UF&ICP-MS)
Nano Ag	Ag nanoparticles	The measured total Ag minus total Ag^+^
