# Supplementary material for: Exposure Medium: Key in Identifying Free Ag+ as the Exclusive Species of Silver Nanoparticles with Acute Toxicity to Daphnia magna
Source: Sci Rep. 2015 Apr 10;5:9674. doi: 10.1038/srep09674 (PMC4392358; doi:10.1038/srep09674)
Supplement: Supplementary Information [file srep09674-s1.pdf]

Supporting Information

for

Exposure Medium: Key in Identifying Free Ag^+ as the Exclusive Species of Silver Nanoparticles with Acute Toxicity to *Daphnia magna*

Mo-Hai Shen[†], Xiao-Xia Zhou[†], Xiao-Ya Yang[†, ‡], Jing-Bo Chao[§], Rui Liu[†], and Jing-Fu Liu[†,*]

[†]State Key Laboratory of Environmental Chemistry and Ecotoxicology, Research Center for Eco-Environmental Sciences, Chinese Academy of Sciences, P. O. Box 2871, Beijing 100085, China.

[‡]School of the Environment, Jiangsu University, Zhenjiang 212013, China.

[§]Chemical Metrology and Analytical Science Division, National Institute of Metrology, Beijing 100013, China.

This Supporting Information includes a total of 11 pages (including this page) with four sections for experimental, references, 4 tables, and 4 figures.

* Corresponding author: E-mail: jfliu@rcees.ac.cn

Tel.: +86-10-62849192; Fax: +86-10-62849192

EXPERIMENTAL

Synthesis of AgNPs. To prepare the polyvinylpyrrolidones (PVP) coated small sized AgNP ($AgNP_{PVP10}$), 0.375 g PVP (molecular weight, MW=58,000) was dissolved in 70 mL ultrapure water. Then, 2.25 mL of 0.1 mol L^{-1} $AgNO_3$ was added into the solution, and the mixture was stirred for 5 min before 2.75 mL of ice-cold $NaBH_4$ (0.08 mol L^{-1}) was added all at once. The mixture was stirred in ice-cold bath for futher 30 min.[1] The PVP coated AgNPs were washed using 100 kDa Ultra-15 centrifugal filter and centrifuged for 30 min at 5000 rpm by a Sigma 3-18 K centrifuge (St. Louis, MO). The residue upon the filter was redispersed in additional 10 mL ultrapure water for further purification. The wash procedure was repeated for three times.

To synthesize PVP coated large sized AgNP ($AgNP_{PVP28}$), 10 g PVP (MW=10,000) was dissolved in 75 mL ethylene glycol, 400 mg $AgNO_3$ was added into the solution. The suspension was stirred until complete dissolution of $AgNO_3$ was achieved. The solution was then heated up to 120 ℃ at a constant rate of 1 ℃ min^{-1}, and was kept at 120 ℃ for 1 h allowing the reaction fully proceeded. The colloidal dispersion was cooled in tap water, at the end of the reaction period, until the system reached room temperature.[2] The AgNPs can be separated easily from the ethylene glycol after adding acetone (V:V, 1:4) followed by centrifugation for 25 min at 7000 rpm. Then, the collected AgNPs were washed by adding ultrapure water followed by centrifugation for 25 min at 7000 rpm. The water-wash procedure was repeated for three times. The obtained AgNPs was collected and washed by ultrapure water using 100 kDa Ultra-15 centrifugal filter and centrifuged for 30 min at 3000 rpm. The residue upon the filter was redispersed in additional 10 mL ultrapure water for further purification. The ultrafiltration wash procedure was repeated for three times.

Stock suspensions of the two AgNPs were prepared by redispersion of the PVP coated AgNPs with ultrapure water. The transmission electron microscopy (TEM) samples of AgNPs were prepared by loading 10 μL aliquots of suspensions at proper concentrations onto ultrathin carbon-coated copper grid, and drying at room temperature. The size distribution of AgNPs were determined using Nano Measurer 1.2 (Informer Technologies, Inc.) and Gaussian fitting, with at least 133 particles counted from multi-

TEM-images in $AgNP_{PVP}$ cases. Since the sizes of commercial $AgNP_{CIT}$s were relatively uniform, the graphs of statistic distribution of nanoparticle sizes were not presented anymore. The stock suspensions were stored in dark at 4 ℃.

REFERENCES

1 Yang, X. *et al.* Mechanism of silver nanoparticle toxicity is dependent on dissolved silver and surface coating in caenorhabditis elegans. *Environ. Sci. Technol.* **46**, 1119-1127 (2012).

2 Silvert, P. Y., HerreraUrbina, R., Duvauchelle, N., Vijayakrishnan, V. & Elhsissen, K. T. Preparation of colloidal silver dispersions by the polyol process Part 1-Synthesis and characterization. *J. Mater. Chem.* **6**, 573-577 (1996).

Table S1. The calculated 8-h median lethal concentrations ($LC50_{8\text{-h}}$) and concentration range of lethal effect of AgNPs and $AgNO_3$ suspensions to *Daphnia manga* (p<0.05).

AgNP type	$LC50_{8\text{-h}}$ as nominal total Ag (μg L^{-1})	Concentration range of lethal effect (μg L^{-1})	
		Letha probability (1%)	Letha probability (99%)
$AgNP_{PVP10}$	5.04 ± 0.84	1.05	24.04
$AgNP_{PVP28}$	16.37 ± 2.08	4.70	57.00
$AgNP_{CIT10}$	6.31 ± 0.69	1.95	20.40
$AgNP_{CIT20}$	39.47 ± 1.40	28.78	54.13
$AgNP_{CIT40}$	102.59 ± 5.80	49.95	210.72
$AgNP_{CIT60}$	116.59 ± 5.68	75.82	179.27
$AgNP_{CIT100}$	144.25 ± 9.47	73.49	283.12
Ag^+ (in $AgNO_3$)	0.78 ± 0.10	0.34	1.79

Table S2. Measured concentrations of different Ag species and total Ag in AgNP and $AgNO_3$ suspensions equivalent to $LC50_{8\text{-h}}$.

AgNP type	Free Ag^+ ($\mu g\ L^{-1}$)	Total Ag^+ ($\mu g\ L^{-1}$)	Dissolved Ag ($\mu g\ L^{-1}$)	Nano Ag ($\mu g\ L^{-1}$)	Measure Total Ag ($\mu g\ L^{-1}$)
$AgNP_{PVP10}$	0.44 ±0.13	1.40 ±0.07	1.41 ±0.04	3.28 ±0.19	4.68 ±0.12
$AgNP_{PVP28}$	0.42 ±0.06	1.07 ±0.02	1.42 ±0.09	12.94 ±0.15	14.01 ±0.12
$AgNP_{CIT10}$	0.44 ±0.07	1.05 ±0.01	1.12 ±0.02	4.65 ±0.02	5.70 ±0.02
$AgNP_{CIT20}$	0.37 ±0.02	1.32 ±0.04	0.91 ±0.05	34.92 ±2.13	36.25 ±2.09
$AgNP_{CIT40}$	0.44 ±0.09	2.01 ±0.24	1.15 ±0.02	83.78 ±1.04	85.79 ±0.79
$AgNP_{CIT60}$	0.43 ±0.06	2.62 ±0.15	1.58 ±0.09	107.36 ±1.33	109.98 ±1.18
$AgNP_{CIT100}$	0.41 ±0.05	2.15 ±0.11	0.99 ±0.09	124.35 ±6.96	126.51 ±6.85
Ag^+ (in $AgNO_3$)	0.40 ±0.05	NA[a]	NA	NA	0.44 ±0.01

[a]NA= not available

63 **Table S3**. Proportions of different Ag species in AgNP suspensions at $LC50_{8\text{-}h}$ as total Ag.

AgNP type	Free Ag^+ (%)	Total Ag^+ (%)	Dissolved Ag (%)	Nano Ag (%)
$AgNP_{PVP10}$	9.31 ± 2.72	29.89 ± 1.46	30.11 ± 0.85	70.11 ± 3.98
$AgNP_{PVP28}$	2.98 ± 0.40	7.62 ± 0.17	10.13 ± 0.66	92.38 ± 1.04
$AgNP_{CIT10}$	7.63 ± 1.26	18.43 ± 0.05	19.73 ± 0.39	81.57 ± 0.39
$AgNP_{CIT20}$	1.02 ± 0.06	3.64 ± 0.10	2.52 ± 0.14	96.36 ± 5.87
$AgNP_{CIT40}$	0.52 ± 0.10	2.34 ± 0.28	1.34 ± 0.03	97.66 ± 1.21
$AgNP_{CIT60}$	0.39 ± 0.05	2.38 ± 0.14	1.44 ± 0.08	97.62 ± 1.21
$AgNP_{CIT100}$	0.32 ± 0.04	1.70 ± 0.08	0.78 ± 0.07	98.30 ± 5.50

Table S4. The analysis of variance (ANOVA) results (95% confidence interval)

Ag species	Significance (p)[a]		
	Among AgNPs	*vs* free Ag^+ in $AgNO_3$[b]	*vs* measured total Ag in $AgNO_3$[b]
Free Ag^+	0.91	0.66	0.64
Total Ag^+	6.9E-10	1.3E-03	1.7E-03
Dissolved Ag	3.5E-08	7.2E-06	1.3E-05

[a] The $p>0.05$ indicates no significant difference, $p<0.05$ indicates significant difference.

[b] The free Ag^+ (0.40 ± 0.05) by ISE and measured total Ag (0.44 ± 0.01) by ICP-MS after digestion in $AgNO_3$ solution had no significant difference with each other ($p=0.27$).

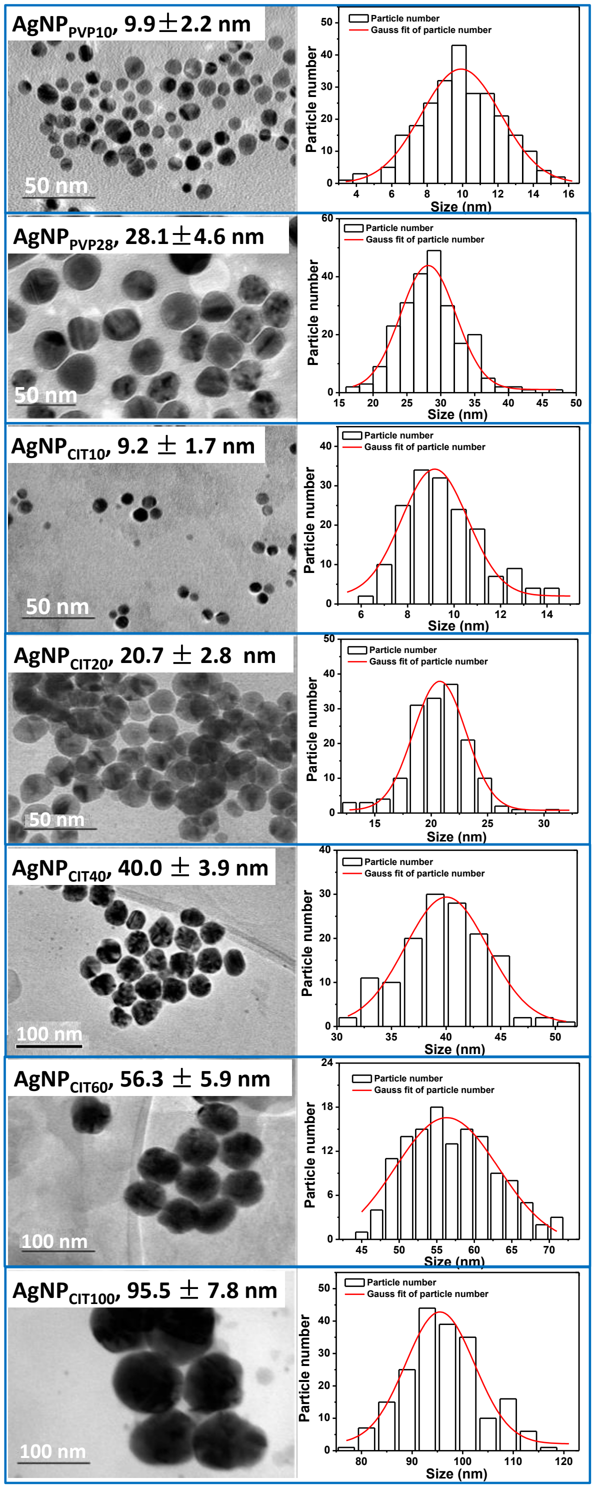

Figure S1. TEM images of AgNPs. Size distribution and primary size determination of each AgNP were performed by measuring the diameter of more than 133 nanoparticles in multi-TEM images.

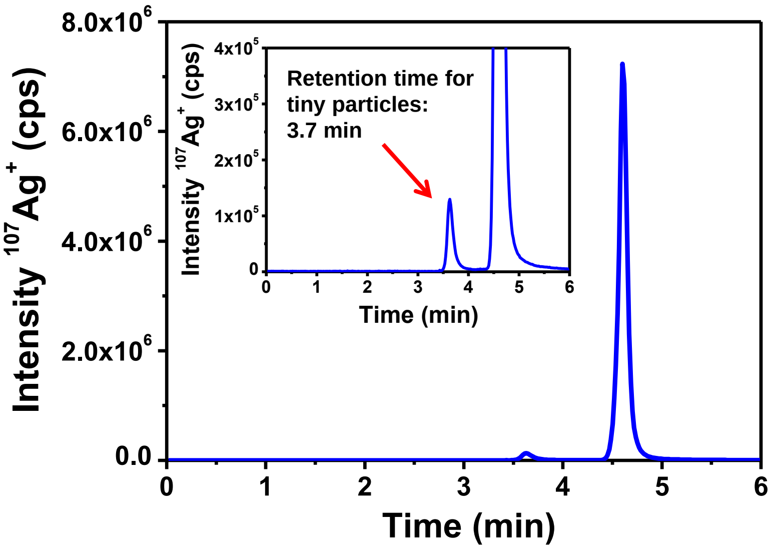

Figure S2. LC-ICP-MS separation and identification of tiny particles in dissolved Ag species of $AgNP_{PVP10}$ separated by centrifugal ultrafiltration. The appearance of the peak at ~3.7 min indicates the existence of tiny Ag particles. The large peak at ~4.6 min was assigned to Ag^{+}.

Measurement of Free Ag^+ by Silver Ion-Selective Electrode (ISE) in AgNPs and $AgNO_3$ (Figure S3-4)

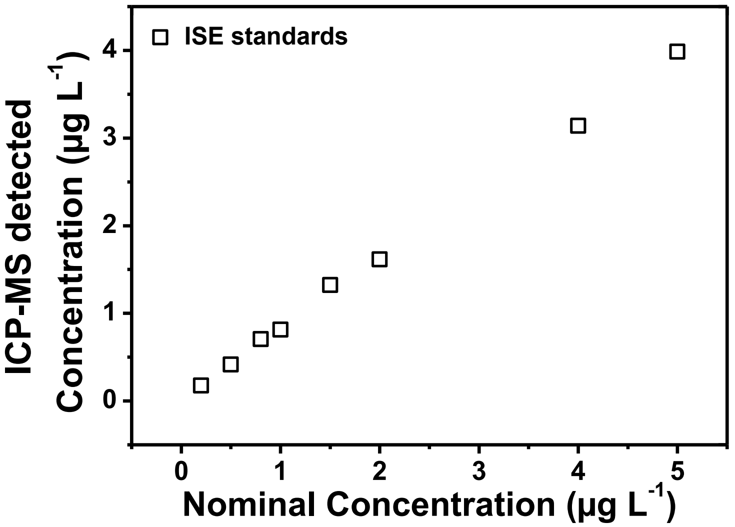

Figure S3. Concentrations of ISE standard measured by ICP-MS. Given the predictable sorption of Ag^+ to glass beakers after the preparation of standard solutions, the Ag^+ concentrations of ISE standards were determined by ICP-MS simultaneously with the ISE standards' measurement. The ICP-MS detected concentrations were used to plotted against potential differences to prepare the calibration curve for ISE measurement of free Ag^+ in AgNPs and $AgNO_3$.

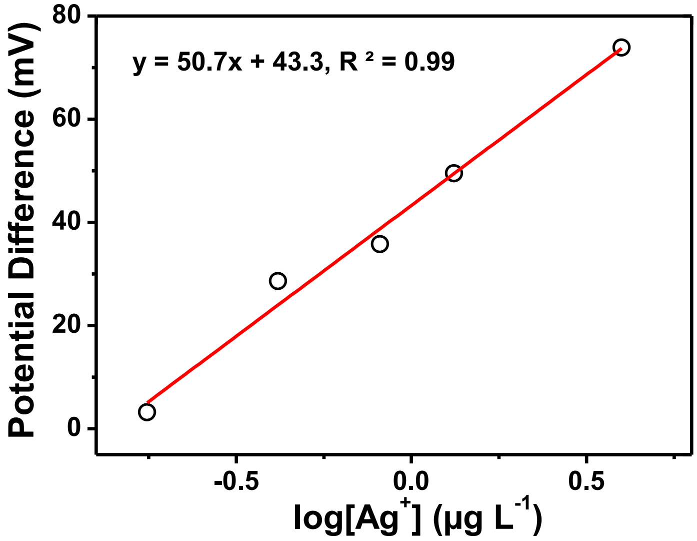

Figure S4. Representative calibration of ISE measurement in the range of 0.18-3.99 μg L^{-1} Ag^+ (in $AgNO_3$, detected by ICP-MS) with a slope of 50.7 mV/log [Ag^+]. The free Ag^+ (0.40 ± 0.05 μg L^{-1}) by ISE and total Ag (0.44 ± 0.01 μg L^{-1}) measured by ICP-MS after digestion in $AgNO_3$ solution equivalent to $LC50_{8\text{-}h}$ had no significant difference with each other (p=0.27). As the measured total Ag is equivalent to free Ag^+ in $AgNO_3$ solution, the agreement between the free Ag^+ by ISE and total Ag by ICP-MS suggested the free Ag^+ results determined by ISE method is credible.
